# Family-Centered Care: How Close Do We Get When Talking to Parents of Children Undergoing Diagnosis for Autism Spectrum Disorders?

**DOI:** 10.1007/s10803-020-04765-0

**Published:** 2020-11-02

**Authors:** Lynnea Myers, Sharon M. Karp, Mary S. Dietrich, Wendy S. Looman, Melanie Lutenbacher

**Affiliations:** 1 Present Address: Gustavus Adolphus College, 800 College Avenue West, Saint Peter, MN 56082 USA; 2 Vanderbilt University School of Nursing, 461 21st Avenue South, Nashville, TN USA; 3 Vanderbilt University School of Medicine, 1161 21st Avenue South, Nashville, TN USA; 4 University of Minnesota School of Nursing, 308 SE Harvard Street, Minneapolis, MN USA; 5 Karolinska Institutet Center of Neurodevelopmental Disorders (KIND), Stockholm, Sweden

**Keywords:** Parents, Autism spectrum disorders, Family-centered care, Communication, Diagnosis

## Abstract

Autism spectrum disorder (ASD) affects 1:59 children, yet little is known about parents’ perceptions of family-centered care (FCC) during the diagnostic process leading up to diagnosis. This mixed-methods study explored key elements of FCC from 31 parents of children recently diagnosed with ASD using parallel qualitative and quantitative measures. Parents rated highly their receipt of FCC and discussed ways providers demonstrated FCC. However, the majority of parents indicated that the period when their child was undergoing diagnosis was stressful and reported symptoms of depression and anxiety. The study points to ways in which health care providers can enhance FCC provided to families when a child is undergoing ASD diagnosis.

**Electronic supplementary material:**

The online version of this article (10.1007/s10803-020-04765-0) contains supplementary material, which is available to authorized users.

Numerous professional, national, and international organizations emphasize the importance of patient and family-centered care (FCC) to achieve quality healthcare outcomes (Committee on Hospital Care and Institute for Patient- and Family-Centered Care [IPFCC] 2012; Institute of Medicine 2001; Medical Home Initiatives for Children with Special Needs Project Advisory Committee 2002; Robert Wood Johnson Foundation, n.d.). Inherent in FCC is the development of a mutually beneficial partnership between families and health care providers (Committee on Hospital Care and IPFCC 2012). Effective communication between parents and providers is critical to FCC and an expectation of quality pediatric health care (Levetown and AAP Committee on Bioethics 2008). Many parents and providers, however, continue to describe difficulties with their communication interactions, especially parents of children undergoing diagnosis for autism spectrum disorders (Brogan and Knussen 2003; Crane et al. 2016; Moh and Magiati 2012; Siklos and Kerns 2007). Several months or years may pass from the time when children are first identified with concerns related to their development until they receive a diagnosis of ASD (Zuckerman et al. 2015). This is a period when parents may have frequent interactions with a multitude of health care providers before receiving a diagnosis of ASD (Rosenberg et al. 2008; Simpson et al. 2003). It is unclear how parents perceive FCC and communication specifically with various health care providers during this time. Additionally, past studies (e.g., Abbott et al. 2013; Brogan and Knussen 2003; Gaspar de Alba and Bodfish 2011) have explored communication between parents and providers at the time ASD is diagnosed but have failed to examine the communication that occurs in the months and years before an ASD diagnosis is obtained.

Strong evidence indicates that the elements of FCC are critical to effective interactions between parents and providers, especially during the sometimes prolonged diagnostic period for ASD. FCC promotes listening, honoring, and respecting the child and family, sharing information, collaborating, and recognizing and building on the strengths of the child and family (Committee on Hospital Care and IPFCC 2012). Studies report that parents want to receive FCC elements during the diagnostic process, including being listened to and asked about their questions/concerns (Abbott et al. 2013; Brogan and Knussen 2003); being able to share and receive information (Crane et al. 2016; Osborne and Reed 2008; Siklos and Kerns 2007); and collaborating with providers (Moh and Magiati 2012). Most studies in ASD have examined perceptions of FCC solely from a quantitative perspective (Brogan and Knussen 2003; Crane et al. 2016; Gaspar de Alba and Bodfish 2011; Moh and Magiati 2012; Siklos and Kerns 2007). Several studies (e.g., Cheak-Zamora and Farmer 2015; Kuo et al. 2011; Magana et al. 2012; Montes and Halterman 2011) have used a standardized measure of FCC from the National Survey of Children’s Health (and the National Survey of Children with Special Health Care Needs (NS-CSHCN). A majority of families of children with ASD in these studies report high-quality FCC in their interactions with providers. These studies have used quantitative methods to explore levels of FCC. This method fails to capture what parents mean when only a rating scale is used and it does not address other important factors such as parental mental health.

Parents of children with ASD may experience significant stress during the diagnostic process (Brogan and Knussen 2003; Costa et al. 2017; Crane et al. 2016; Siklos and Kerns 2007). This heightened stress may influence how parents either communicate or receive information (Osborne et al. 2008). Similarly, emerging research suggests parental mental health issues may affect perceptions of FCC and parent-provider interactions. For example, in a study of 195 caregivers of young children with asthma (Fagnano et al. 2012), researchers found parents who reported symptoms of depression were less likely to report the health care provider as reassuring and encouraging, asking the family about how they managed the asthma, or giving information. Parental anxiety may be an additional issue to consider when exploring elements of FCC. Abbott et al. (2013) found that parents appreciated when providers were aware of the parent’s anxiety and tried to help them feel at ease. Although research exploring the influence of parent mental health on elements of FCC is relatively new, evidence suggests parental stress, depression, and anxiety may impact the communication process that occurs with the child’s health care provider.

The primary aim of this study was to use a mixed methods design to gain a richer understanding of parents’ FCC experiences with health care providers during the time period their child was first identified with concerns related to ASD to when they actually received a diagnosis (this time period is referred to hereafter as “diagnostic process”). A secondary aim of this study was to explore the relationship between parental mental health issues and perceived levels of FCC during the diagnostic process.

## Methods

This exploratory study used a mixed method design with parallel qualitative and quantitative methods and measures. The qualitative method included one-on-one interviews, followed by completion of a structured, on-line quantitative survey which paralleled the interview questions and included brief questions related to parental mental health issues.

### Participants

Parents in the US were included in the study if they (1) had a child between the ages of 18 months to 6 years who they self-reported had received a diagnosis of ASD from a health care provider in the prior 12 months; (2) spoke English; (3) were over the age of 18 years; (4) were the child’s legal guardian and their primary caretaker; and, (5) had access to a phone and/or computer. Parents were recruited via an informational flyer that was sent out from a variety of recruitment sources: ASD parent groups (i.e., Facebook parent groups and a clinic-based ASD support group), advocacy organizations (e.g., societies for ASD and individuals with disabilities), diagnostic resource centers, a research listserv, and a volunteer registry created by several academic institutions and supported by the US National Institutes of Health (i.e., ResearchMatch). The target sample for the study was 20–30 parents, which was based on final sample sizes that achieved saturation of themes in other qualitative studies exploring communication between parents and providers (e.g., Howe 2014; Howe et al. 2015; Jimenez et al. 2013; Shannon 2004; Sices et al. 2009; Stille et al. 2010, 2013; Watson et al. 2006). A total of 95 parents expressed interest in participating in the study; 31 met the eligibility criteria. Individuals were primarily excluded if their child was older than 6 years of age, the child received the ASD diagnosis more than 12 months prior, or they did not have a child diagnosed with ASD. The recruitment and data collection for the study took place January to March 2017.

### Measures

#### Structured Interview Guide

A structured interview guide was developed for this study (Supplemental File 1) that included nine questions exploring the parents’ experiences during the time the diagnostic process that were similar to those from the National Survey of Children’s Health (Centers for Disease Control and Prevention 2015b) and National Survey of Children with Special Health Care Needs (NS-CSHCN 2010) related to FCC and parent-provider communication.

Three nurse researchers with expertise in pediatrics and two researchers with expertise in ASD reviewed and revised the interview guide to enhance feasibility, clarity, completeness, and fit with study purpose. The guide was then pilot tested with four parents who met the inclusion criteria for the study. These parents were asked to respond to each interview question and then provide feedback related to the questions. One parent made suggestions for minor edits to enhance the clarity of a few questions and the prompts included in the final interview guide. The interviews took approximately 20–30 min to complete.

#### Survey

The online survey included four sections: FCC; timing of diagnosis; parent depression and anxiety; and parent stress. The survey took less than 5 min for parents to complete.

##### Family-Centered Care

Five questions from the Centers for Disease Control and Prevention (2015b) and the NS-CSHCN (2010) (Centers for Disease Control and Prevention 2015a, b) were used to assess FCC: provider spends time with the parent; provider elicits concerns and listens carefully; provider sensitive to the family’s values and customs; parent reports getting needed information; and parent feels like a partner in the care of their child (Supplemental File 2). The questions were slightly modified from their original form by removing the introductory clause in each question (“In the last 12 months”) to encourage broader reflection on the diagnostic process (which may have been longer than 12 months). Parents rated their level of experience for each question on FCC using a four-point Likert scale (1 = never, 2 = sometimes, 3 = usually, 4 = always). The questions have previously demonstrated concurrent validity (Bethell et al. 2004) and good internal consistency (Drummond et al. 2012). The alpha coefficient for internal consistency reliability of these items in this study was 0.80.

##### Timing of Diagnosis

Two questions were used to explore timing of diagnosis of ASD for child. These included (1) at what age were concerns about the child’s development first noticed and by whom and, (2) at what age did the parent first learn their child had a diagnosis of ASD.

##### Depression and Anxiety

Four questions comprising the Patient Health Questionnaire (PHQ-4) (Kroenke et al. 2009) were used to assess how often parents experienced problems related to depression and anxiety. This measure was selected due to its brevity with four questions, as well as reported validity and reliability. The measure has good construct validity with two factors: depression and anxiety (Kroenke et al. 2009) and good internal consistency (alpha = 0.81; Khubchandani et al. 2016; Löwe et al. 2010). Parents were asked to respond to questions on the scale by reflecting back on the period in which their child was in the process of being diagnosed with ASD. Responses to all four items on the PHQ-4 were summed together to create a total score with a range between 0 and 12. Scores were interpreted as follows: 0–2 = normal; 3–5 = mild symptoms of depression or anxiety; 6–8 = moderate symptoms depression or anxiety; and 9–12 = severe symptoms depression or anxiety. The Cronbach’s alpha for the scale in this study was 0.80.

##### Stress

Parent stress was assessed using four-point Likert-scale question asking parents to rate the level of stress (1 = not at all stressful to 4 = very stressful) that they felt This same question was previously used in a large study by Crane et al. (2016) exploring parents’ experience with ASD diagnosis in the UK. The Cronbach’s alpha for the scale in this study was 0.89.

### Procedure

Study procedures were approved by the (blinded for review) Institutional Review Board (IRB). Interested parents contacted the principal investigator (PI) to learn more about the study and to determine their eligibility. Informed consent was obtained verbally at the start of the interview process, which were conducted via web-based video conference or telephone and audio-recorded with consent. After the interview, parents completed the online survey electronically through REDCap, a secure web based application designed to build surveys and manage databases (Harris et al. 2009). Parents received a $50 Amazon gift card for participation in the study.

### Data Analysis

Quantitative and qualitative data were analyzed using a concurrent design (Fetters et al. 2013) where initial analysis of each type of data took place concurrently, but independently. Subsequently, results from the qualitative analysis were merged with results from the quantitative analysis to help interpret and discuss the findings. Specific procedures used for the both qualitative and quantitative analyses are outlined below.

#### Qualitative Analysis

Audio recordings of interviews were transcribed verbatim. Two trained coders (PI and an experienced, qualitative researcher with a master’s degree in social psychology), with oversight from a senior researcher with expertise in qualitative analysis, used directed content analysis with the qualitative data. Each statement made by participants in the study was treated as a separate quote, and each quote was coded using a hierarchical coding system. The hierarchical coding system was developed using an iterative, inductive-deductive approach based on the study questions, interview guide, study framework, and a preliminary assessment of the first several interviews. In total, 52 codes were developed and used to code 1429 quotes from parents. The two coders independently reviewed all quotes, assigned codes, and established inter-rater agreement. The two coders met on a weekly basis to resolve all discrepancies in coding until all transcripts were coded, thereby resulting in complete agreement on the codes assigned to each quote. At the conclusion of the coding process, the PI (Coder 1) used an iterative process to read the quotes by categories and codes and summarize their main points. Next, to ensure credibility and confirmability of the findings, both coders, along with the senior researcher, met over several weeks to discuss the summary and major themes. After major themes were confirmed, illustrative quotes from the interviews were selected to support the themes, along with statistics citing the frequency of coded quotes supporting each theme.

#### Quantitative Analysis

Frequencies and percentages were used to summarize the parents’ responses to the items in the online survey. Means and standard deviations were calculated for normally-distributed continuous variables; median and IQR for skewed distributions. For the five questions regarding FCC used from the national surveys (i.e., NSCH/CSHCN), a continuous variable representing FCC was constructed indicating the number of questions that a parent reported as “usually” or “always” receiving. Values for the FCC variable ranged from “0” (no FCC elements received) to “5” (all five FCC elements received). No imputation of missing data was needed as parents completed all survey items in this study. SPSS, version 24, was used to conduct the quantitative analyses. An alpha of 0.05 was used for determining statistical significance.

## Results

### Sample

Thirty-one parents (representing 31 children diagnosed with ASD) participated in the study. Demographic characteristics of both parents and their children are summarized in Table 1. The average parental age was 34.5 years (SD = 7.2) and average age of their children was 3.9 years (SD = 1.3) at the time of the interview. Most of the parents were white (74.2%) and female (90.3%), while the majority of the children were also white (67.7%), but male (71%). Six of the 31 families reported having at least one other child diagnosed with ASD and two additional families reported having another child with special needs. Most parents reported being married or living with a significant other (80.6%) and had at least some college education or more (93.5%). Over a third (35.5%) of parents reported having public health insurance for their child (e.g., Medicaid) and more than half of the parents (51.6%) reported a family income less than $60,000 annually. Parents were recruited from the entirety of the US, though a majority (48.4%) were recruited from Minnesota and Tennessee, as these were the states where the researchers had greatest access to ASD parent groups, advocacy organizations, and diagnostic resource centers.

**Table 1:** Descriptive statistics of participants and their children (n = 31)

Characteristic	N (%)
Parent gender	
Male	3 (9.7)
Female	28 (90.3)
Child gender	
Male	22 (71.0)
Female	9 (29.0)
Parent ethnicity	
Not Hispanic or Latino	30 (96.8)
Hispanic or Latino	1 (3.2)
Parent race	
Asian	4 (12.9)
Asian and White	1 (3.2)
Black or African American	3 (9.7)
White	23 (74.2)
Child ethnicity	
Not Hispanic or Latino	30 (96.8)
Hispanic or Latino	1 (3.2)
Child race	
American Indian or Alaska Native	1 (3.2)
American Indian and White	1 (3.2)
Asian	4 (12.9)
Asian and White	1 (3.2)
Black or African American	2 (6.5)
Black or African American and White	1 (3.2)
White	21 (67.7)
Type of insurance (child)	
Private	16 (51.6)
Public	11 (35.5)
Military	1 (3.2)
Private and public	1 (3.2)
Public and military	2 (6.5)
Annual household income (USD)	
$0–20,000	3 (9.7)
$20,001–40,000	8 (25.8)
$40,001–60,000	5 (16.1)
$60,001 or more	15 (48.4)
Parent marital status	
Married or living with significant other	25 (80.6)
Separated	1 (3.2)
Divorced	1 (3.2)
Single	4 (12.9)
Parent highest educational level	
High school education	2 (6.5)
Some college, no degree	8 (25.8)
Associate’s degree	5 (16.1)
Bachelor’s degree	8 (25.8)
Master’s degree or higher	8 (25.8)

	Mean (SD) and minimum/maximum
Parent age (years)	34.5 (7.2), 19–48
Child age (years)	3.9 (1.3), 2.0–6.0
Child age when diagnosed with ASD (years)	3.3 (1.2), 1.6–6.0

	Median (IQR) and minimum/maximum
Child age when concerns first identified (years)	1.3 (1.0, 1.8), 0.5–4.0
Time from first concerns to diagnosis (years)	1.5 (0.9, 3.0), 0.3–5.0

Parents reported communicating with a range of 2–6 health care providers from the time when they first identified concerns about their child to when they received a diagnosis of ASD, including physicians (i.e., family practice physicians, pediatricians, developmental pediatricians, psychiatrists, neurologists), nurse practitioners, nurses, psychologists, and speech and occupational therapists. The majority of parents (67.7%) reported that the main provider they communicated with during the diagnostic period was a pediatrician, other physician, or nurse practitioner.

### Quantitative Results

Summaries of the parent responses to the questions regarding FCC from the NSCH/NS-CSHCN survey items are shown in Table 2. About half of parents reported receiving all five elements of FCC (54.8%, n = 17), while 25.8% (n = 8) reported receiving three or four, and 19.4% (n = 6) reported receiving fewer than three. Although the majority of parents reported “usually” or “always” receiving each of these five FCC elements from their health care providers, nearly all (96.8%, n = 30) of the respondents endorsed the provider usually or always being sensitive to the family’s values and customs. The least frequently endorsed FCC element was getting needed information from the provider (67.7%, n = 24).

**Table 2:** Parent perceptions of elements of family-centered care using the national survey of children’s health/national survey of children with special health care needs (n = 31)

Key element	N (%)
How often child's doctors and other health care providers spend enough time with him/her	
Never	3 (9.7)
Sometimes	5 (16.1)
Usually	14 (45.2)
Always	9 (29.0)
How often child's doctors and other health care providers listen carefully to parent	
Never	3 (9.7)
Sometimes	5 (16.1)
Usually	9 (29.0)
Always	14 (45.2)
How often were child’s doctors and other health care providers sensitive to family's values and customs	
Sometimes	1 (3.2)
Usually	8 (25.8)
Always	22 (71.0)
How often did parents get specific information you needed from child's doctors and other health care providers	
Never	2 (6.5)
Sometimes	8 (25.8)
Usually	13 (41.9)
Always	8 (25.8)
How often did child's doctors or other health care providers help parent feel like a partner in child’s care	
Never	2 (6.5)
Sometimes	6 (19.4)
Usually	9 (29.0)
Always	14 (45.2)
Number of key elements parents report “usually” or “always” receiving from health care providers	
0	1 (3.2)
1	3 (9.7)
2	2 (6.5)
3	4 (12.9)
4	4 (12.9)
5	17 (54.8)

The median PHQ-4 score was 5.6 (IQR = 2, 8), a score that represents moderate symptoms of depression or anxiety. The median rating of stress during the diagnostic process was 3 (IQR = 2, 4), with 22 parents (71%) reporting the diagnostic process for their child was either “quite stressful” or “very stressful” (Table 3). Associations between the number of FCC elements a parent reported receiving with parental stress and PHQ-4 scores are shown in Table 4. A statistically significant inverse correlation was observed between parent reports of the number of FCC elements received and stress (r_s_ = − 0.43, p = 0.016). That is, as the number of elements decreased, reported stress increased.

**Table 3:** Parent mental health characteristics during the time of diagnosis (n = 31)

Mental health characteristic	Mean (SD)
Parental stress overall score	3.06 (.89)
	N (%)
Not at all stressful	1 (3.2)
Not very stressful	8 (25.8)
Quite stressful	10 (32.3)
Very stressful	12 (38.7)

	Median (25th and 75th interquartile range)
PHQ-4 (anxiety and depression) overall score	6 (2, 8)
	N (%)
No symptoms of depression or anxiety	8 (25.8)
Mild symptoms of depression or anxiety	6 (19.4)
Moderate symptoms of depression or anxiety	12 (38.7)
Severe symptoms of depression or anxiety	5 (16.1)

**Table 4:** Spearman correlations between elements of family-centered care and parent mental health characteristics (n = 31)

Characteristic	Number of elements of FCC
Parental Stress	− .43 (0.016)
PHQ-4	− .17 (0.364)

Values in the cells are *r*_*s*_ (p-value)

### Qualitative Results with Parallel Quantitative Results

Through the interviews, parents identified both effective and ineffective provider behavior related to the following FCC elements: (1) elicits concerns and listens; (2) sensitive to values and customs; (3) knowledgeable and gives information; (4) treats parent as a partner; and (5) spends enough time with parent. To highlight these findings, summaries of each FCC element are presented followed by quotes from parent interviews. In addition, severity of symptoms of anxiety and depression on the PHQ-4 and parent level of stress during the diagnostic process are presented. The number of quotes taken from coded interviews supporting each theme are listed.

#### Elicits Concerns and Listens

Many parents reported that providers asked about and were sensitive to their concerns about their child’s development (25 quotes) and answered their questions right away or got back to them if they did not immediately know the answers (7 quotes). Similarly, nearly 75% of parents reporting providers “usually” or “always” elicit concerns and listen. Parents described how providers welcomed their concerns and that they were not left alone with their concerns, but put at ease. Providers not only asked questions, but also took the time to listen to the parent and clarify what was heard (49 quotes). Provider behaviors that suggested to parents that providers were listening included making good eye contact, asking questions, taking notes, validating concerns, and not interrupting the parent. A quote from a parent who rated providers as “always” eliciting concerns and listening, reported severe symptoms of anxiety and depression, and described the diagnostic process as “very stressful” stated:

Okay. I would say for [diagnostic clinic] that yes, they listened to us and whatever questions that we had they made sure to answer them completely and they would ask a couple times, "Are you sure you don't have anything else?”

Despite most parents describing effective ways in which providers approached eliciting parental concerns and listening, several parents (25 quotes) described ways they did not feel listened to, including feeling their concerns were disregarded/doubted and feeling their providers were not taking the time to listen to the specific details related to their child. Some parents discussed how they felt their child might have received a diagnosis or services earlier in the process if the providers had listened more to the parents’ concerns. A parent who rated providers as “never” eliciting their concerns or listening, had moderate symptoms of anxiety and depression and described the diagnostic process as “very stressful”:

I wish they had listened more….and really just don’t doubt me. I think a parent definitely knows more about their child…they know something is changing or something just seems off. I wish they had listened more.

#### Sensitive to Values and Customs

Almost all parents (97%) reported “usually” or “always” feeling that providers were sensitive to their values and customs. Many parents reported the main way providers illustrated this was through respect. For example, a few parents addressed how providers respected their decisions to withhold vaccinations. However, some parents did not feel that they had any particular values or customs that were necessary to consider in the care of their child. This is the perspective of a parent who rated providers as “usually” sensitive to values and customs, reported normative levels for symptoms of anxiety and depression, and described the diagnostic process as “not very stressful”:

…I mean she was really just very respectful. I mean she didn't push anything on me…

#### Knowledgeable and Gives Information

Parents generally described providers as knowledgeable about ASD (27 quotes), with almost 68% of parents reporting “usually” or “always” receiving information. Parents described many types of information and referrals to resources that they received from health care providers during the process of obtaining a diagnosis (44 quotes). A few parents mentioned they received written summaries from visits and how helpful these summaries were to review after the visits. Other parents talked about providers giving them specific recommendations for treatment, therapies, or referrals. A parent who rated “always” receiving information, reported normative levels for symptoms of anxiety and depression, and described the diagnostic process as “not at all stressful” stated:

She [psychologist] provided us with a five-page report, very detailed, of every assessment she used and those scores. Then the overall scores, as well as medical and developmental history, and the interview process for them. Then at the end she listed recommendations of what to continue with and was able to supply me with resources in the area that I could get in touch with.

Some parents, however, found providers lacked knowledge and weren’t able to provide information (10 quotes). Concerns centered around lack of knowledge to diagnose ASD in younger children or in girls, in particular, and in understanding the various ways ASD may present, especially in high-functioning children. Parent concerns were most often directed towards the primary care provider who they felt was unfamiliar with early detection of ASD as this was not their specialty. This was in contrast to parent experience with the providers or therapists experienced with diagnosing and working, respectively, with children with ASD.

Many parents noted additional information they wished they had received from providers (20 quotes), but only learned about later in the process. Examples included referrals to other resources, services in the community such as social services or parent groups, suggestions on how to work with the child at home, and a checklist on the steps to take following a diagnosis to arrange appropriate services for their child. Some parents discussed complete lack of information related to their child’s diagnosis or next steps, causing them to find information entirely on their own. Parents specifically noted their concern about providers not giving information as they were trying to understand what was going on with their child. A few parents who had other children previously diagnosed with ASD assumed some information was not given to them because they were thought to already have knowledge of the disorder. A parent who rated providers “sometimes” gave information, reported normative levels for symptoms of anxiety and depression, described the diagnostic process as “quite stressful.” When she was asked about the types of information received from providers during the diagnostic process, she stated:

Not much at all actually…. I think that at some point, I would hope that when you go through a medical diagnosis then you would get more…I feel like most of what I’ve learnt, I’ve looked up myself, I’ve read myself.

#### Treats Parent as a Partner

Most parents felt that they were partners in the care of their child (28 quotes), which aligned with the survey responses where nearly 75% of parents reported “usually” or “always” feeling like a partner. Parents mentioned how providers gave them suggestions on what they could do at home with their child to enhance the child’s development and/or checked with the parent whether or not these suggestions would work for their family. Parents also appreciated providers who asked about their opinions regarding the next steps for their children in terms of treatment, therapies, or referrals. A parent who rated “always” feeling treated as a partner on the survey, reported normative levels for symptoms of anxiety and depression, and described the diagnostic process as “quite stressful” said:

They always make sure that I was okay with things and that it was okay for them to do certain things and they always kind of came back to you and made sure that you were okay with what the next thing that they were going to do. So they would explain it to the child, but at the same time, they're kind of asking permission.

About half of the parents who reported “never” or only “sometimes” being treated like a partner reported that they did not feel trusted or regarded as an expert in the care of their child. This was contrary to their feeling that they had legitimate concerns about their child’s development and wanted to have those concerns acknowledged. In fact, parents reported in 42 quotes ways in which providers were dismissive of their concerns. These parents discussed how they wished providers would take their concerns about their child’s development seriously early on and make referrals for further exploration into those concerns, especially since parents spent the most time with their child and knew their child best.

Some parents reported not feeling like partners in the process of the diagnosis for their child (9 quotes). One parent commented she had to rely solely on the decisions of the provider for next steps as their insurance did not cover referrals unless made by the primary care provider. A few parents mentioned the challenge of talking with providers at their level to feel like they were a partner in the care of their child. One parent who rated “sometimes” being treated as a partner on the survey, reported moderate symptoms of anxiety and depression, and described the diagnostic process as “very stressful”:

I felt like I was in it alone.

#### Enough time

Parents commented positively on providers who took time to fully address their concerns and who did not make them feel rushed. Nearly 75% of parents reported that the provider “usually” or “always” spent enough time with their child. Many parents discussed how appreciative they were of the time the provider spent with them, especially in the context of a busy clinic setting. The following quote is from a parent who rated “always” having enough time with the provider, reported severe symptoms of anxiety and depression, and described the diagnostic process as “quite stressful”:

She was fine spending as much time with us as we needed to feel comfortable. She's always been very generous with her time despite the busyness of the practice.

Parents described providers not having enough time to spend with them (26 quotes) in a busy primary care setting to discuss concerns or questions or that the visit felt rushed. For the parents who specifically responded “never” or only “sometimes” having enough time with the provider, many indicated those seen earlier in the diagnostic process, usually primary care providers. A parent who rated “sometimes” having enough time with the provider, reported moderate symptoms of anxiety and depression, and described the diagnostic process as “very stressful”:

It's always so extremely rushed like how fast can we get all the details we need and get you in and out of here… A major barrier was just the lack of time, always feeling rushed.

## Discussion

This unique mixed methods study combining an online survey with an interview contributes to a better understanding of parental perspectives of FCC and communication with health care providers during the diagnostic process for ASD. It also examined relationship between these perceptions and the parent’s self-reported levels of anxiety, depression, and stress during this time period. Although most parents reported they received at least some elements of FCC during the diagnostic process, there was variability across parental experiences. The majority of parents reported interacting with 2–6 types of providers, making it difficult to establish which types of providers offered effective and/or ineffective FCC experiences.

Using parallel forms of items in interview and survey questions offered an opportunity to assess the congruence between the two forms of measurement and to gain a richer insight to the experience. For most, survey ratings and qualitative comments were congruent in reflecting a parent’s perspective. This further validates the use of the FCC items from the NSCH and NS-CSHCN with other similar parents.

Findings from our work highlight how emotionally and mentally challenging the diagnostic process for ASD can be, even when parents feel that providers are delivering FCC and including them in the diagnostic process. Perhaps the most important finding is that the majority of parents reported moderate symptoms of depression and anxiety and that the diagnostic process for ASD was stressful. Reed and Osborne (2018), in a sample of 120 mothers who had a child recently diagnosed with ASD, found higher levels of anxiety and depression, along with prolonged grief and poorer health status, in the mothers who had unresolved reactions to the diagnostic process compared with mothers with resolved reactions. In our study, nearly 55% of parents reported experiencing moderate to severe symptoms of anxiety and depression during their child’s diagnosis. Over 70% reported the diagnostic process to be “quite stressful” or “very stressful”. Using quantitative measures, we were able to see that higher levels of reported stress were significantly associated with reports of fewer elements of FCC received. Study findings underscore the importance of further exploration of parental mental health, particularly levels of stress, during the diagnostic process for ASD.

### Elements of FCC

Similar to previous studies (Abbott et al. 2013; Guerrero et al. 2010, 2011; Halfon et al. 2004), the majority of parents in our study felt providers elicited their concerns about their child’s development and listened to them; however, some parents reported that providers were often dismissive and did not listen to or elicit parents’ concerns. This finding is similar to what was reported in a recent systematic review (Legg and Tickle 2019) exploring the diagnostic experiences of parents in the United Kingdom (U.K.). This finding and other current evidence support the importance parents place on having their concerns sought and heard during the diagnostic process for ASD. These provider actions have the potential to greatly enhance the communication process so that parents feel that the process is more collaborative.

Similar to a study of 40 parents in the United Kingdom (UK) (Hackett et al. 2009), nearly all parents in our study reported feeling that providers were sensitive to their values and customs and primarily described being treated with respect by the provider. Even if a provider does not fully understand the cultural background or values of families they serve, consistent, respectful communication seems to make parents feel as if the provider is sensitive to their values and customs. Being respected by the provider is a critical element to a trusting relationship with a provider, which in turn, has the potential to lead to enhanced health behaviors, quality of life, and satisfaction with the care received (Birkhauer et al. 2017).

Although some parents in our study found provider knowledge and the specific information very helpful, other parents reported being concerned about providers; inadequate levels of knowledge and information. In a similar study that explored experiences of parents of 16 children diagnosed with ASD (Sansosti et al. 2012), parents listed specific information they wished they had received during the diagnostic process, Our findings suggest that details about the child’s diagnosis and recommended next steps interventions or referrals were important components of information that were not always received. Not surprisingly, Sansosti et al. found that parents were more satisfied with information received from providers who had special training in ASD. In 2009, Golnik and colleagues assessed the perceptions of 539 physicians regarding their level of competency to care for children with ASD. Pediatric and family practice doctors reported a low level of competency to care for children with ASD, including their inability to provide resources or to develop a trusting and satisfactory relationship with the parents. They also identified a need for more education related to caring for children with ASD. Our findings suggest this need for improvement remains an issue and primary care providers need increased knowledge about diagnosis, early intervention, treatment, and support services for ASD.

While many of our study’s parents reported that they received needed information from providers, they also reported they wanted more information about support groups, social services, and therapies. These findings are consistent with findings from other studies (e.g., Osborne and Reed 2008; Tait et al. 2016; Wong et al. 2017). On the other hand, a few parents in our study expressed feeling overwhelmed at times with the information they received, which Abbott et al. (2013) also found in interviews with parents of children diagnosed with ASD in the UK. This finding highlights the importance of provider assessment of parental readiness to receive information. Some parents need to receive brief amounts of information, while others need more comprehensive information. With the parent-provider relationship during the diagnostic process often being long term with the child’s main provider, the process of delivering information at the appropriate time and in an appropriate way is critical. Accomplishing this may be as straightforward as a checklist with essential steps to take and key information to provide following diagnosis, as recommended by one parent in our study.

Most parents in this study reported feeling like a partner in their child’s care, though some felt they were not valued as the expert on their child’s development. This finding is similar to large survey studies using weighted samples of children from various racial and ethnic groups with a diagnosis of ASD such as the 2005/2006 and 2009/2010 NS-CSHCN (Magana et al. 2015), While most of our parents reported feeling like a partner in their child’s care, some felt they were not valued as the expert on their child’s development. A unique aspect of this study was the opportunity for parents to specifically describe ways in which their providers made them feel like a partner in the care of their child. Most often, examples offered included a reciprocal process where providers made suggestions on what parents could do at home to help their child, while asking parents if these suggestions were helpful, in addition to asking parents’ opinions about next steps for treatment, therapies, or referrals. Conversely, a few parents also discussed their concerns about not being treated like experts in the care of their child and not being seen as partners in the diagnostic process. Our findings underscore how simple steps to engage parents, such as checking in with the parent and asking the parent’s opinion, may be very effective means of helping the parent become a partner and recognized as an expert on their child.

Most parents reported providers spent enough time with their child, but others, particularly when referencing primary care providers, said they did not have enough time, similar to findings in Sansosti et al. (2012). Several parents thought the lack of time was due to the busy nature of the health care system, particularly primary care. Although it is difficult for an individual provider to change what may be a more systemic issue, it highlights the importance of potential provider influence on health policy and encouraging innovative health care delivery. Robins (2020) called for more "humanity" in health care through authentic conversations and listening to patients and families. Policies that provide enhanced health care reimbursement for time communicating with and counseling parents may help incentivize providers to spend more time with parents.

### Strengths and Limitations

Strengths of this study included the mixed methods design with saturation of qualitative data, the recent time frame in which parents reported their child had received a diagnosis of ASD, the young age of children, and the use of technology to recruit a geographically diverse sample of parents. The study captured an array of parent experiences during the diagnostic period for ASD, including facilitators and barriers to the communication process between parents and providers. Study limitations included a small sample size, selection bias as parents volunteered to enroll in the study, and limited diversity of parent race, ethnicity, and gender, affecting the ability to generalize findings. Standardized measures and interview questions relied on parent self-report that, in turn, may be associated with recall bias related to difficulty remembering things from the past and/or influenced by social desirability. We also only explored the communication process from the perspective of parents. In addition, with subjects reporting interacting with 2–6 different providers, it was not possible to draw conclusions about which providers at which points in the diagnostic process were perceived as helpful. Future studies with larger samples could also explore the relationship between types of health care providers and FCC and communication during the diagnostic process for ASD. Additionally, investigating providers’ perspectives on limitations or barriers to offering FCC to families of children with ASD would be of interest.

## Conclusion

This study contributes to understanding how parents perceive and experience FCC during the diagnostic process for ASD for their child. Similar to findings of other studies, the majority of parents in this study felt they had received FCC, but also reported high levels of stress. The parents reporting stress identified specific negative experiences including too little time spent with providers, the perceived dismissive nature of some providers about parent concerns, and providers not listening to them. Results contribute to a better understanding of the impact of provider interactions with parents of young children undergoing the diagnostic process for ASD.

## Electronic supplementary material

Below is the link to the electronic supplementary material.

Supplementary file1 (DOCX 139 kb)

Supplementary file2 (DOCX 323 kb)
