# Supplementary material for: Family-Centered Care: How Close Do We Get When Talking to Parents of Children Undergoing Diagnosis for Autism Spectrum Disorders?
Source: J Autism Dev Disord. 2020 Nov 2;51(9):3073–84. doi: 10.1007/s10803-020-04765-0 (PMC8349341; doi:10.1007/s10803-020-04765-0)
Supplement: Supplementary file 1 — Supplementary file1 (DOCX 139 kb) [file 10803_2020_4765_MOESM1_ESM.docx]

Family-Centered Care: How Close Do We Get When Talking to Parents of Children Undergoing Diagnosis for Autism Spectrum Disorders?

*Journal of Autism and Developmental Disorders*

Lynnea Myers^1^, Sharon M. Karp^2^, Mary S. Dietrich^2,3^, Wendy S. Looman^4^, Melanie Lutenbacher^2,3^

Corresponding Author: Dr. Lynnea Myers, Gustavus Adolphus College, lmyers@gustavus.edu

Supplemental File 1

Interview Guide

**Introduction:**

Today’s discussion will focus on your child who was diagnosed with ASD during the last 12 months. I want to learn more about your experiences during the time when (insert child’s name) was first identified with concerns about his/her development to when you received a diagnosis of ASD. I am going to ask you a series of questions about your child’s development and ways in which health care providers (defined as doctors, nurse practitioners, psychologists, therapists, and nurses) communicated with you about (insert child’s name) development and next steps during the time it took to obtain a diagnosis of ASD. For the questions, please reflect on a primary health care provider you worked with during the process as well as other providers you may have encountered during this process. The information you provide will help me and other health care providers better understand the perspective of parents during the diagnostic process for ASD and identify better ways for providers to communicate with parents during this process. There are no anticipated risks for you to participate in this interview.

The interview will take about 30-60 minutes. Please answer the questions however you see appropriate. There are no right or wrong answers to the questions, rather, I am interested in gathering your experiences and opinions. You may choose to refuse to answer a question or to stop the interview at any time, without any penalty. Please ask me for clarification if a question is confusing. I have a few research details I am going to share with you now. All information you provide will be kept anonymous. A random numerical code has been assigned to you and will be linked to your responses. The only exception to maintaining confidentiality and privacy is if I am told something to indicate you may hurt yourself or someone else. In this case, I will need to share that information with appropriate individuals who can help. The interview will be audio-recorded for transcription purposes. If you would like to participate in this study, I ask you to provide consent verbally for your participation in the study with a ‘yes, I would like to participate’, indicating we may proceed.

Questions for Parent (in bold):

1. **Tell me a little bit about how the concern about (insert child’s name) development was first identified?** Prompts: who, when, where, how, etc.
   1. If the parent indicates they first identified the concern: **What did you do once you identified the concern?** Prompts: Who did you talk to? How did you share these concerns with the health care provider? What happened after that?
2. **Thinking back to those first visits where you or your child’s health care provider had initial concerns about your child’s development,**
   1. **how did the provider explain the concerns about your child and his/her development (or if parent had concerns initially) or how did the provider react to your concerns?** Prompt: I heard you say…can you explain that more? Probe: Did the provider directly describe his or her concerns or provide more vague information about his or her concerns?
   2. **what were your thoughts as the provider talked to you about your child’s development?** Prompt: I heard you say…can you explain that more?

The next set of questions will explore your interactions with your child’s health care provider during the diagnostic process from when you first had concerns about your child’s development up to the point when you received a diagnosis of ASD for your child. Often, parents work with one primary provider, but also interact with other providers during the course of obtaining a diagnosis. Was this the case for you? (Yes, No).

If yes, then

1. Can you tell me what type of health care provider the main provider was (e.g., physician, physician’s assistant, nurse practitioner, other nurse like public health nurse, clinic nurse, etc. or other [please describe])? Was the provider male or female? Can you give me an estimation of how many times you interacted with this provider?
2. If you interacted with multiple providers, do you remember how many? What types of health care providers were they? Was the provider male or female?

If no, then

1. Can you tell me what type of provider or providers you interacted? List out each – male/female and estimation of contact times for each.

For the questions below, focus on the main provider (parents can answer generally about other providers also)

1. **Thinking back to how accessible or how much time your child’s health care provider(s) spent with you during the diagnostic process for ASD, was it was enough, too much or not enough time? What kinds of things made you feel that way?**
   1. Prompt: What amount of time did he or she spend per visit? What amount of time did he or she spend over the entire diagnostic process? Probe: If applicable, which health care provider(s) are your referring to?
2. **Did you feel your child’s health care provider was listening to you during the diagnostic process for ASD? What kinds of experiences made you feel that way?** Probe: Did you feel your concerns were heard or acknowledged? If applicable, which health care provider(s) are your referring to?
3. **Was your child’s health care provider sensitive to your family's values and customs during the diagnostic process for ASD? What kinds of experiences made you feel that way?** Probe: If applicable, which health care provider(s) are your referring to?
4. **What kinds of information did you get from your child’s health care provider during the diagnostic process for ASD? How often did you get the information you needed?**
   1. Follow-up: If you did not get the information you needed, please describe why you felt that way and what additional information you would have liked to receive. Did you feel like you got enough information or was it too little? Probe: If applicable, which health care provider(s) are your referring to?
5. **How did your child’s health care provider help you feel like a partner in the care of your child during the diagnostic process for ASD? What kinds of experiences made you feel that way?**
   1. Follow-up: If the provider did not help you feel like a partner in the care of your child, please describe why you felt that way. Probe: If applicable, which health care provider(s) are your referring to?
6. **What did you see as barriers to communication with your child’s health care provider during the diagnostic process for ASD?** Prompt: What, if anything, made it difficult to talk to your child’s health care provider? Probe: If applicable, which health care provider(s) are your referring to?
7. **What did you see as facilitators or things that were positive to communication with your child’s health care provider during the diagnostic process for ASD?** Prompt: What, if anything, made it easy or positive to talk with your child’s health care provider? Probe: If applicable, which health care provider(s) are your referring to?

**Is there anything else you would like to share about the communication you had with your child’s health care provider during the diagnostic process for ASD or do you have recommendations for providers as they communicate with other families that have concerns about their child’s development or a diagnosis of ASD?** Probe: If applicable, which health care provider(s) are your referring to?
