# Supplementary material for: Family-Centered Care: How Close Do We Get When Talking to Parents of Children Undergoing Diagnosis for Autism Spectrum Disorders?
Source: J Autism Dev Disord. 2020 Nov 2;51(9):3073–84. doi: 10.1007/s10803-020-04765-0 (PMC8349341; doi:10.1007/s10803-020-04765-0)
Supplement: Supplementary file 2 — Supplementary file2 (DOCX 323 kb) [file 10803_2020_4765_MOESM2_ESM.docx]

Family-Centered Care: How Close Do We Get When Talking to Parents of Children Undergoing Diagnosis for Autism Spectrum Disorders?

*Journal of Autism and Developmental Disorders*

Lynnea Myers^1^, Sharon M. Karp^2^, Mary S. Dietrich^2,3^, Wendy S. Looman^4^, Melanie Lutenbacher^2,3^

Corresponding Author: Dr. Lynnea Myers, Gustavus Adolphus College, lmyers@gustavus.edu

Supplemental File 2

Quantitative Survey on Family-Centered Care
